# Supplementary material for: Smooth Interpolating Curves with Local Control and Monotone Alternating Curvature
Source: Comput Graph Forum. 2022 Oct 6;41(5):25–38. doi: 10.1111/cgf.14600 (PMC9827861; doi:10.1111/cgf.14600)
Supplement: Supplementary file 1 — Supplement Material [file CGF-41-25-s001.zip › Local-Smooth-Interpolating-MonoCurvature/extern/clothoids/docs/api-cpp/program_listing_file_Clothoids_Line.hxx.html]

Program Listing for File Line.hxx — Clothoids v2.0.9

### Navigation

- index
- toc
- Clothoids »
- Program Listing for File Line.hxx

# Program Listing for File Line.hxx¶

↰ Return to documentation for file (`Clothoids/Line.hxx`)

```
/*--------------------------------------------------------------------------*\
 |                                                                          |
 |  Copyright (C) 2017                                                      |
 |                                                                          |
 |         , __                 , __                                        |
 |        /|/  \               /|/  \                                       |
 |         | __/ _   ,_         | __/ _   ,_                                |
 |         |   \|/  /  |  |   | |   \|/  /  |  |   |                        |
 |         |(__/|__/   |_/ \_/|/|(__/|__/   |_/ \_/|/                       |
 |                           /|                   /|                        |
 |                           \|                   \|                        |
 |                                                                          |
 |      Enrico Bertolazzi                                                   |
 |      Dipartimento di Ingegneria Industriale                              |
 |      Universita` degli Studi di Trento                                   |
 |      email: enrico.bertolazzi@unitn.it                                   |
 |                                                                          |
\*--------------------------------------------------------------------------*/


namespace G2lib {

  /*\
   |   _     _
   |  | |   (_)_ __   ___
   |  | |   | | '_ \ / _ \
   |  | |___| | | | |  __/
   |  |_____|_|_| |_|\___|
  \*/

  class LineSegment : public BaseCurve {

    friend class CircleArc;
    friend class PolyLine;

    real_type m_x0;
    real_type m_y0;
    real_type m_theta0;

    real_type m_c0;
    real_type m_s0;
    real_type m_L;

  public:

    #include "BaseCurve_using.hxx"

    //explicit
    LineSegment()
    : BaseCurve(G2LIB_LINE)
    , m_x0(0)
    , m_y0(0)
    , m_theta0(0)
    , m_c0(1)
    , m_s0(0)
    , m_L(0)
    {}

    //explicit
    LineSegment( LineSegment const & s )
    : BaseCurve(G2LIB_LINE)
    { copy(s); }

    explicit
    LineSegment( BaseCurve const & C );

    explicit
    LineSegment(
      real_type _x0,
      real_type _y0,
      real_type _theta0,
      real_type _L
    )
    : BaseCurve(G2LIB_LINE)
    , m_x0(_x0)
    , m_y0(_y0)
    , m_theta0(_theta0)
    , m_c0(cos(_theta0))
    , m_s0(sin(_theta0))
    , m_L(_L)
    {}

    void
    copy( LineSegment const & c ) {
      m_x0     = c.m_x0;
      m_y0     = c.m_y0;
      m_theta0 = c.m_theta0;
      m_c0     = c.m_c0;
      m_s0     = c.m_s0;
      m_L      = c.m_L;
    }

    LineSegment const & operator = ( LineSegment const & s )
    { copy(s); return *this; }

    // . . . . . . . . . . . . . . . . . . . . . . . . . . . . . . . . . . . . .

    real_type
    length() const override
    { return m_L; }

    real_type
    length_ISO( real_type ) const override
    { return m_L; }

    /*\
     |   _     _
     |  | |__ | |__   _____  __
     |  | '_ \| '_ \ / _ \ \/ /
     |  | |_) | |_) | (_) >  <
     |  |_.__/|_.__/ \___/_/\_\
    \*/

    void
    bbox(
      real_type & xmin,
      real_type & ymin,
      real_type & xmax,
      real_type & ymax
    ) const override;

    void
    bbox_ISO(
      real_type   offs,
      real_type & xmin,
      real_type & ymin,
      real_type & xmax,
      real_type & ymax
    ) const override;

    /*\
     |  _    _   _____    _                _
     | | |__| |_|_   _| _(_)__ _ _ _  __ _| |___
     | | '_ \ '_ \| || '_| / _` | ' \/ _` | / -_)
     | |_.__/_.__/|_||_| |_\__,_|_||_\__, |_\___|
     |                               |___/
    \*/

    void
    bbTriangles(
      std::vector<Triangle2D> & tvec,
      real_type                 max_angle = Utils::m_pi/6, // 30 degree
      real_type                 max_size  = 1e100, // unused
      int_type                  icurve    = 0
    ) const override {
      real_type xmin, ymin, xmax, ymax;
      this->bbox( xmin, ymin, xmax, ymax );
      real_type xc = (xmax+xmin)/2;
      real_type yc = (ymax+ymin)/2;
      real_type nx = (ymax-ymin)/100;
      real_type ny = (xmin-xmax)/100;
      if ( xmax > xmin || ymax > ymin ) {
        Triangle2D t( xmin, ymin, xmax, ymax, xc+nx, yc+ny, 0, 0, icurve );
        tvec.push_back( t );
      } else {
        UTILS_ERROR(
          "LineSegment bbTriangles found a degenerate line\n"
          "bbox = [ xmin={}, ymin={}, xmax={}, ymax={} ] max_angle={} max_size={}\n",
          xmin, ymin, xmax, ymax, max_angle, max_size
        );
      }
    }

    void
    bbTriangles_ISO(
      real_type                 offs,
      std::vector<Triangle2D> & tvec,
      real_type                 max_angle = Utils::m_pi/6, // 30 degree
      real_type                 max_size  = 1e100, // unused
      int_type                  icurve    = 0
    ) const override {
      real_type xmin, ymin, xmax, ymax;
      this->bbox_ISO( offs, xmin, ymin, xmax, ymax );
      real_type xc = (xmax+xmin)/2;
      real_type yc = (ymax+ymin)/2;
      real_type nx = (ymax-ymin)/100;
      real_type ny = (xmin-xmax)/100;
      if ( xmax > xmin || ymax > ymin ) {
        Triangle2D t( xmin, ymin, xmax, ymax, xc+nx, yc+ny, 0, 0, icurve );
        tvec.push_back( t );
      } else {
        UTILS_ERROR(
          "LineSegment bbTriangles found a degenerate line\n"
          "bbox = [ xmin={}, ymin={}, xmax={}, ymax={} ]\n"
          "offs={} max_angle={} max_size={}\n",
          xmin, ymin, xmax, ymax, offs, max_angle, max_size
        );
      }
    }

    void
    bbTriangles_SAE(
      real_type                 offs,
      std::vector<Triangle2D> & tvec,
      real_type                 max_angle = Utils::m_pi/6, // 30 degree
      real_type                 max_size  = 1e100,
      int_type                  icurve    = 0
    ) const override {
      this->bbTriangles_ISO( -offs, tvec, max_angle, max_size, icurve );
    }

    /*\
     |   ____             _          _______           _
     |  | __ )  ___  __ _(_)_ __    / / ____|_ __   __| |
     |  |  _ \ / _ \/ _` | | '_ \  / /|  _| | '_ \ / _` |
     |  | |_) |  __/ (_| | | | | |/ / | |___| | | | (_| |
     |  |____/ \___|\__, |_|_| |_/_/  |_____|_| |_|\__,_|
     |              |___/
    \*/

    real_type tx_Begin() const override { return m_c0; }
    real_type ty_Begin() const override { return m_s0; }
    real_type tx_End()   const override { return m_c0; }
    real_type ty_End()   const override { return m_s0; }

    real_type nx_Begin_ISO() const override { return -m_s0; }
    real_type ny_Begin_ISO() const override { return m_c0; }
    real_type nx_End_ISO()   const override { return -m_s0; }
    real_type ny_End_ISO()   const override { return m_c0; }

    real_type xBegin() const override { return m_x0; }
    real_type yBegin() const override { return m_y0; }
    real_type xEnd()   const override { return m_x0+m_L*m_c0; }
    real_type yEnd()   const override { return m_y0+m_L*m_s0; }

    real_type
    xBegin_ISO( real_type offs ) const override
    { return m_x0+offs*nx_Begin_ISO(); }

    real_type
    yBegin_ISO( real_type offs ) const override
    { return m_y0+offs*ny_Begin_ISO(); }

    real_type
    xEnd_ISO( real_type offs ) const override
    { return xEnd()+offs*nx_Begin_ISO(); }

    real_type
    yEnd_ISO( real_type offs ) const override
    { return yEnd()+offs*ny_Begin_ISO(); }

    /*\
     |  _   _          _
     | | |_| |__   ___| |_ __ _
     | | __| '_ \ / _ \ __/ _` |
     | | |_| | | |  __/ || (_| |
     |  \__|_| |_|\___|\__\__,_|
    \*/

    real_type
    theta( real_type ) const override
    { return m_theta0; }

    real_type
    theta_D( real_type ) const override
    { return 0; }

    real_type
    theta_DD( real_type ) const override
    { return 0; }

    real_type
    theta_DDD( real_type ) const override
    { return 0; }


    /*\
     |  _____                   _   _   _
     | |_   _|   __ _ _ __   __| | | \ | |
     |   | |    / _` | '_ \ / _` | |  \| |
     |   | |   | (_| | | | | (_| | | |\  |
     |   |_|    \__,_|_| |_|\__,_| |_| \_|
    \*/

    real_type tx( real_type ) const override { return m_c0; }
    real_type ty( real_type ) const override { return m_s0; }
    real_type tx_D( real_type ) const override { return 0; }
    real_type ty_D( real_type ) const override { return 0; }
    real_type tx_DD( real_type ) const override { return 0; }
    real_type ty_DD( real_type ) const override { return 0; }
    real_type tx_DDD( real_type ) const override { return 0; }
    real_type ty_DDD( real_type ) const override { return 0; }

    // . . . . . . . . . . . . . . . . . . . . . . . . . . . . . . . . . . . . .

    void
    tg( real_type, real_type & tx, real_type & ty ) const override
    { tx = m_c0; ty = m_s0; }

    void
    tg_D( real_type, real_type & tx_D, real_type & ty_D ) const override
    { tx_D = ty_D = 0; }

    void
    tg_DD( real_type, real_type & tx_DD, real_type & ty_DD ) const override
    { tx_DD = ty_DD = 0; }

    void
    tg_DDD( real_type, real_type & tx_DDD, real_type & ty_DDD ) const override
    { tx_DDD = ty_DDD = 0; }

    // . . . . . . . . . . . . . . . . . . . . . . . . . . . . . . . . . . . . .

    real_type
    X( real_type s ) const override
    { return m_x0+s*m_c0; }

    real_type
    Y( real_type s ) const override
    { return m_y0+s*m_s0; }

    real_type
    X_D( real_type ) const override
    { return m_c0; }

    real_type
    Y_D( real_type ) const override
    { return m_s0; }

    real_type
    X_DD( real_type ) const override
    { return 0; }

    real_type
    Y_DD( real_type ) const override
    { return 0; }

    real_type
    X_DDD( real_type ) const override
    { return 0; }

    real_type
    Y_DDD( real_type ) const override
    { return 0; }

    // . . . . . . . . . . . . . . . . . . . . . . . . . . . . . . . . . . . . .

    void
    eval(
      real_type   s,
      real_type & x,
      real_type & y
    ) const override {
      x = m_x0+s*m_c0;
      y = m_y0+s*m_s0;
    }

    void
    eval_D(
      real_type,
      real_type & x_D,
      real_type & y_D
    ) const override {
      x_D = m_c0;
      y_D = m_s0;
    }

    void
    eval_DD(
      real_type,
      real_type & x_DD,
      real_type & y_DD
    ) const override {
      x_DD = 0;
      y_DD = 0;
    }

    void
    eval_DDD(
      real_type,
      real_type & x_DDD,
      real_type & y_DDD
    ) const override {
      x_DDD = 0;
      y_DDD = 0;
    }

    /*\
     |         __  __          _
     |   ___  / _|/ _|___  ___| |_
     |  / _ \| |_| |_/ __|/ _ \ __|
     | | (_) |  _|  _\__ \  __/ |_
     |  \___/|_| |_| |___/\___|\__|
    \*/

    real_type
    X_ISO( real_type s, real_type offs ) const override
    { return m_x0 + s*m_c0 + offs*nx_Begin_ISO(); }

    real_type
    Y_ISO( real_type s, real_type offs ) const override
    { return m_y0 + s*m_s0 + offs*ny_Begin_ISO(); }

    real_type
    X_ISO_D( real_type, real_type ) const override
    { return m_c0; }

    real_type
    Y_ISO_D( real_type, real_type ) const override
    { return m_s0; }

    real_type
    X_ISO_DD( real_type, real_type ) const override
    { return 0; }

    real_type
    Y_ISO_DD( real_type, real_type ) const override
    { return 0; }

    real_type
    X_ISO_DDD( real_type, real_type ) const override
    { return 0; }

    real_type
    Y_ISO_DDD( real_type, real_type ) const override
    { return 0; }

    // . . . . . . . . . . . . . . . . . . . . . . . . . . . . . . . . . . . . .

    void
    eval_ISO(
      real_type   s,
      real_type   offs,
      real_type & x,
      real_type & y
    ) const override {
      x = m_x0 + s*m_c0 + offs*nx_Begin_ISO();
      y = m_y0 + s*m_s0 + offs*ny_Begin_ISO();
    }

    void
    eval_ISO_D(
      real_type,
      real_type,
      real_type & x_D,
      real_type & y_D
    ) const override {
      x_D = m_c0;
      y_D = m_s0;
    }

    void
    eval_ISO_DD(
      real_type,
      real_type,
      real_type & x_DD,
      real_type & y_DD
    ) const override {
      x_DD = y_DD = 0;
    }

    void
    eval_ISO_DDD(
      real_type,
      real_type,
      real_type & x_DDD,
      real_type & y_DDD
    ) const override {
      x_DDD = y_DDD = 0;
    }

    /*\
     |  _                        __
     | | |_ _ __ __ _ _ __  ___ / _| ___  _ __ _ __ ___
     | | __| '__/ _` | '_ \/ __| |_ / _ \| '__| '_ ` _ \
     | | |_| | | (_| | | | \__ \  _| (_) | |  | | | | | |
     |  \__|_|  \__,_|_| |_|___/_|  \___/|_|  |_| |_| |_|
    \*/

    void
    translate( real_type tx, real_type ty ) override
    { m_x0 += tx; m_y0 += ty; }

    void
    rotate( real_type angle, real_type cx, real_type cy ) override;

    void
    reverse() override;

    void
    changeOrigin( real_type newx0, real_type newy0 ) override
    { m_x0 = newx0; m_y0 = newy0; }

    void
    scale( real_type sc ) override
    { m_L *= sc; }

    void
    trim( real_type s_begin, real_type s_end ) override {
      m_x0 += m_c0 * s_begin;
      m_y0 += m_s0 * s_begin;
      m_L   = s_end - s_begin;
    }

    /*\
     |      _ _     _
     |   __| (_)___| |_ __ _ _ __   ___ ___
     |  / _` | / __| __/ _` | '_ \ / __/ _ \
     | | (_| | \__ \ || (_| | | | | (_|  __/
     |  \__,_|_|___/\__\__,_|_| |_|\___\___|
    \*/


    int_type
    closestPoint_ISO(
      real_type   qx,
      real_type   qy,
      real_type & x,
      real_type & y,
      real_type & s,
      real_type & t,
      real_type & dst
    ) const override;

    int_type
    closestPoint_ISO(
      real_type   qx,
      real_type   qy,
      real_type   offs,
      real_type & x,
      real_type & y,
      real_type & s,
      real_type & t,
      real_type & dst
    ) const override;

    void
    info( ostream_type & stream ) const override
    { stream << "LineSegment\n" << *this << '\n'; }

    // . . . . . . . . . . . . . . . . . . . . . . . . . . . . . . . . . . . . .
    // . . . . . . . . . . . . . . . . . . . . . . . . . . . . . . . . . . . . .
    // . . . . . . . . . . . . . . . . . . . . . . . . . . . . . . . . . . . . .

    void
    build(
      real_type x0,
      real_type y0,
      real_type theta0,
      real_type L
    ) {
      m_x0     = x0;
      m_y0     = y0;
      m_theta0 = theta0;
      m_c0     = cos(theta0);
      m_s0     = sin(theta0);
      m_L      = L;
    }

    void
    build_2P(
      real_type _x0,
      real_type _y0,
      real_type _x1,
      real_type _y1
    );

    void
    build_2P( real_type const p0[2], real_type const p1[2] )
    { build_2P( p0[0], p0[1], p1[0], p1[1] ); }

    void
    p1p2( real_type p1[2], real_type p2[2] ) const {
      p1[0] = m_x0;
      p1[1] = m_y0;
      p2[0] = m_x0+m_L*m_c0;
      p2[1] = m_y0+m_L*m_s0;
    }

    bool
    intersect(
      LineSegment const & S,
      real_type         & s1,
      real_type         & s2
    ) const;

    bool
    intersect_ISO(
      real_type           offs,
      LineSegment const & S,
      real_type           S_offs,
      real_type         & s1,
      real_type         & s2
    ) const;

    void
    intersect(
      LineSegment const & LS,
      IntersectList     & ilist,
      bool                swap_s_vals
    ) const {
      real_type s1, s2;
      bool ok = this->intersect( LS, s1, s2 );
      if ( ok ) {
        if ( swap_s_vals ) ilist.push_back( Ipair(s2, s1) );
        else               ilist.push_back( Ipair(s1, s2) );
      }
    }

    void
    intersect_ISO(
      real_type           offs,
      LineSegment const & LS,
      real_type           offs_LS,
      IntersectList     & ilist,
      bool                swap_s_vals
    ) const {
      real_type s1, s2;
      bool ok = this->intersect_ISO( offs, LS, offs_LS, s1, s2 );
      if ( ok ) {
        if ( swap_s_vals ) ilist.push_back( Ipair(s2, s1) );
        else               ilist.push_back( Ipair(s1, s2) );
      }
    }

    bool
    collision( LineSegment const & S ) const;

    bool
    collision_ISO(
      real_type           offs,
      LineSegment const & S,
      real_type           S_offs
    ) const;

    /*\
     |   _   _ _   _ ____  ____ ____
     |  | \ | | | | |  _ \| __ ) ___|
     |  |  \| | | | | |_) |  _ \___ \
     |  | |\  | |_| |  _ <| |_) |__) |
     |  |_| \_|\___/|_| \_\____/____/
    \*/

    void
    paramNURBS( int_type & n_knots, int_type & n_pnts ) const;

    void
    toNURBS( real_type * knots, real_type Poly[][3] ) const;

    virtual
    void
    toBS( real_type * knots, real_type Poly[][2] ) const;

    friend
    ostream_type &
    operator << ( ostream_type & stream, LineSegment const & c );

    friend class ClothoidCurve;

  };

}
```

### Quick search

### Table of Contents

- Matlab Interface Manual
- C++ API
- MATLAB API

«
hide menu

menu
sidebar
»

### Navigation

- index
- toc
- Clothoids »
- Program Listing for File Line.hxx

© Copyright 2021, Enrico Bertolazzi and Marco Frego.
Created using Sphinx 4.2.0.
